# Early identification using the referral system prolonged the time to onset for hepatic encephalopathy after diagnosing severe acute liver injury

**DOI:** 10.1038/s41598-020-74466-2

**Published:** 2020-10-14

**Authors:** Keisuke Kakisaka, Yuji Suzuki, Hiroaki Abe, Takuya Watanabe, Kenji Yusa, Hiroki Sato, Yasuhiro Takikawa

**Affiliations:** Division of Hepatology, Department of Internal Medicine, School of Medicine, Iwate Medical University, 1-1-1 Idaidori, Yahaba-cho, Morioka, Iwate 0283694 Japan

**Keywords:** Diseases, Gastroenterology, Risk factors

## Abstract

In 2004, we implemented a referral system for patients with acute liver injury (ALI) based on an established formula that estimates the risk of progression to acute live failure (ALF); however, the benefits of the system for patients with severe acute liver injury (SLI) remain unclear. We have evaluated the clinical significance of the referral system for SLI patients. Patients with ALI/SLI who were consecutively and prospectively listed on the system between 2004 and 2018 were analyzed. Of the 371 ALI/SLI/ALF patients on the system, 124 satisfied the criteria for SLI; 34 of these 124 progressed to SLI after registration. Multivariate analysis using age, sex, AST, ALT, creatinine, total bilirubin, prothrombin, presence of hepatic encephalopathy (HE), and SLI at registration revealed that HE was associated with high mortality. Among the 23 patients who developed HE, five who progressed to SLI after registration showed an increased time to HE development compared with patients who had SLI at the time of registration. However, there was no significant difference in survival time after HE development. We concluded that early identification of SLI patients using the referral system increased the time from SLI diagnosis to HE development.

## Introduction

Acute liver failure (ALF) is characterized by massive hepatocyte loss, which leads to the impairment of protein synthesis and detoxification^1^. The definition of ALF is based on that of “fulminant hepatic failure”, proposed by Trey and Davidson^2^. Originally, fulminant hepatic failure was defined as “a severe liver injury, potentially reversible in nature and with onset of hepatic encephalopathy (HE) within 8 weeks of the first symptoms in the absence of pre-existing liver disease”. It is defined as severe acute liver injury (SLI) that includes ALF without HE, resulting in elevated liver enzymes and coagulopathy. Our previous study revealed that the mortality rate among patients who progressed from acute liver injury (ALI) to SLI/ALF is higher than those with ALI^3^; thus, progression to SLI/ALF is a risk factor of poor prognosis for ALI. Furthermore, previous studies have reported that the mortality rate increases among patients with ALF who develop HE^4–7^.

To predict the development of HE, we established a method to evaluate the probability of HE onset based on age, etiology, and total bilirubin and prothrombin activity; it is referred to as the Japan Hepatic Encephalopathy prediction (JHEP) model^8^. Using this model, we prospectively collected data from patients with ALI in the Northern Tohoku area in Japan, including age, sex, etiology of ALI, laboratory data, treatment, and outcome. We also implemented a treatment strategy for patients who were found to have a high probability of developing HE according to the JHEP model; the detailed procedure has been previously reported^9^. Briefly, patients are managed by physicians in community hospitals after discussion with a hepatologist; management is guided by the probability of HE development as calculated by the JHEP model. For patients with a > 20% probability of developing HE, transfer to the core hospital should be considered as they require careful observation, preemptive therapy for HE, and preparation for liver transplantation. Preemptive therapy was performed when hyperammonemia (more than 70 mg/dL) was found in the patients. The preemptive therapy included administration of lactulose; patients were subsequently treated with rifaximine if hyperammonemia remained. When patients with SLI develop HE, artificial liver support systems such as continuous hemodiafiltration are required. The importance of the referral system in the case of SLI treatment is also recognized in the USA^10^. A review by Dr. Doulberis and his colleagues mentioned that the standard management for SLI indicates transferring the patient to a core center prior to HE development to prepare for liver transplantation^11^. As liver transplantation is the only established curative treatment for ALF^4,11,12^, this is an important procedure for improved prognosis; however, there is currently a shortage of liver donors. Although introduction of presumed consent increases the numbers^13–15^, establishing an adequate organ supply based on appropriate indications for liver transplantation is crucial.

After introducing our JHEP model, the rate of HE development among patients with SLI decreased drastically^16^, indicating effective prevention of HE development via the referral system. As the mortality rate was previously found to be only 11.6% among patients with ALI who progress to SLI/ALF^3^, a more sensitive parameter was required to identify patients who would require liver transplantation. To understand potential indications for liver transplantation in patients with ALF who are listed on the referral system, the risk factors associated with poor prognosis must be investigated. Additionally, the effect of the referral system on the clinical course of such patients needs to be evaluated.

## Results

### Patient characteristics

We recruited 124 participants for this study; 90 who were diagnosed with SLI upon registration on the referral system and 34 who satisfied the SLI criteria during the observation period. Characteristics of the study population, etiology of the liver disease of each patient, and laboratory data at the time of SLI diagnosis are summarized in Table 1. Of the patients with SLI, 23 developed HE and progressed to ALF.

**Table 1:** Patient’s characteristics.

Sex (M: F)	54: 70
Age (range)	57.3 (16–86)
**Etiology**	
AIH (32), DILI (22), HBV (acute^10^, carrier^14^, de novo ^2^), oral (15), undetermined (29)	
**Type of disease**	
Without coma: coma (acute): coma (subacute): LOHF	101: 5: 17: 1
SLI at registration (+ : −)	90: 34
Survival: deceased: liver transplantation	92: 23: 9

	Mean (95% CI)
T-Bil (mg/dL)	11.3 (9.7–12.9)
AST (U/L)	1632 (1244–2021)
ALT (U/L)	1578 (1277–1880)
Cre (mg/dL)	0.77 (0.69–0.86)
PT-INR	2.02 (1.86–2.18)

*AIH* autoimmune hepatitis, *ALT* alanine aminotransferase, *AST* aspartate aminotransferase, *Cre* creatinine, *DILI* drug‐induced liver injury, *HBV* hepatitis B virus, *LOHF* late-onset liver failure, *Oral* hepatitis due to oral infection, *PT-INR* prothrombin time international normalized ratio, *SLI* severe acute liver injury, *T-Bil* total bilirubin.

### Development of HE is a risk factor for poor prognosis in patients with SLI

Binominal logistic regression analysis using laboratory data at the time of SLI diagnosis (aspartate transaminase [AST], alanine transaminase [ALT], creatinine [Cre], total bilirubin [T-Bil], and prothrombin International Normalized Ratio [PT-INR]), sex, age, and development of HE as explanatory variables revealed the development of HE to be the only factor associated with poor prognosis (supplemental Table 1, odds ratio [95% confidential interval]: 260.0 [17.9–3773.4]; *p* < 0.0001). Our previous study reported a decrease in the rate of HE development after implementing the referral system, where preemptive therapy for HE to the ALI patients was suggested to improve the clinical course of patients with SLI. Thus, ALF at the time of registration on the referral system was added to the binominal logistic regression analysis. In this analysis, HE remained the only factor associated with poor prognosis (Table 2).

**Table 2:** Multivariate analysis about prognosis using by disease type at registration, sex, presence of hepatic encephalopathy, age, creatinine, prothrombin, total bilirubin, aspartate aminotransferase and alanine aminotransferase in the 124 SLI patients.

	Odds	95% CI	*p* value
Sex (F)	0.79	0.16–3.82	0.7674
Coma (+)	211.1	15.88–2807.10	< 0.0001
Age	1.04	0.99–1.09	0.1645
Cre	5.71	0.41–79.00	0.1943
PT-INR	1.51	0.37–6.14	0.5664
T-Bil	1.03	0.96–1.10	0.4211
ALT	1.00	0.99–1.01	0.1816
AST	1.00	0.99–1.01	0.6553
SLI at registration	2.71	0.44–16.47	0.2031

*ALT* alanine aminotransferase, *AST* aspartate aminotransferase, *Cre* creatinine, *PT-INR* prothrombin time international normalized ratio, *SLI* severe acute liver injury, *T-Bil* total bilirubin.

### Early identification of SLI patients using the referral system prolonged the time to onset for HE after ALF development

**Figure 1:**
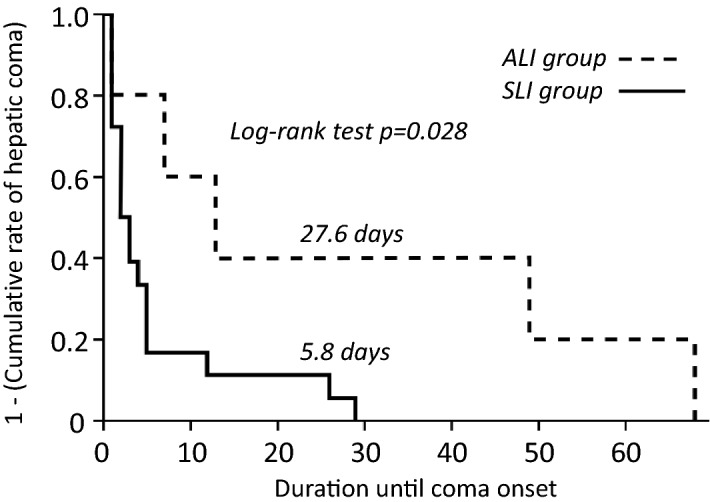
Graphs of the cumulative rate of development of hepatic encephalopathy after diagnosis of acute liver failure. *SLI* severe acute liver injury, *ALI* acute liver injury.

Evaluation of the chronological changes in patients with ALF revealed the mean period to HE development to be 10.6 days after ALF diagnosis. We divided the patients with ALF and HE into two groups; those diagnosed with SLI at the time of registration (the SLI group; n = 18), and those who progressed from ALI at registration to ALF after registration (the ALI group; n = 5). Comparing the laboratory data at the time of SLI diagnosis between these groups (Table 3) revealed ALT and PT-INR to be higher in the SLI group. The cumulative rate of HE development after SLI diagnosis is illustrated in Fig. 1. The mean time to onset of HE after diagnosis was significantly longer in the ALI than SLI group (log-rank test, *p* = 0.028).

**Table 3:** Comparison of patients’ characteristics among the ALI and the SLI groups in patients who progressed to ALF.

	ALI group (n = 5)	SLI group (n = 18)	
Sex (M: F)	1: 4	12: 6	n.s
Age (range)	63 (52–85)	59 (27–82)	n.s
**Etiology**			
AIH, DILI, HBV (acute, carrier, de novo), oral, undetermined	1: 1: 0: 1: 0: 0: 2	3: 2: 3: 4: 2: 1: 3	n.s
**Type of disease**			
Acute: subacute: LOHF	1: 3: 1	4: 14: 0	n.s
Prognosis (deceased: liver transplantation: survival)	2: 2: 1	12: 6: 0	n.s

	Mean (95% CI)	Mean (95% CI)	
T-Bil (mg/dL)	17.6 (8.4–26.8)	13.6 (10.5–16.7)	n.s
AST (U/L)	407 (139–676)	1703 (785–2622)	n.s
ALT (U/L)	524 (174–873)	2188 ( 1077–3298)	*p* = 0.0188
Cre (mg/dL)	0.98 (0.06–2.04)	1.15 ( 1.66–0.67)	n.s
PT-INR	1.58 ( 1.50–1.67)	3.36 (2.59–4.13)	*p* = 0.0046

Wilcoxon/Kruskal–Wallis test was performed for comparison among continuous values.* AIH* autoimmune hepatitis, *ALT* alanine aminotransferase, *AST* aspartate aminotransferase, *Cre* creatinine, *DILI* drug‐induced liver injury, *HBV* hepatitis B virus, *LOHF* late-onset liver failure, *Oral* hepatitis due to oral infection, *PT-INR* prothrombin time international normalized ratio, *SLI* severe acute liver injury, *T-Bil* total bilirubin.

### Early identification of SLI patients using the referral system does not prolong transplant-free survival time after development of HE

**Figure 2:**
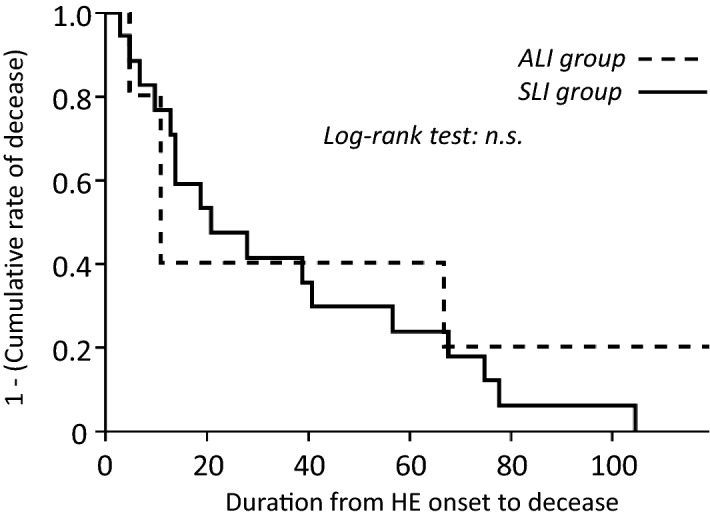
Cumulative rate of transplant-free survival in patients with acute liver failure. *SLI* severe acute liver injury, *ALI* acute liver injury.

The referral system had a positive effect on the time to HE development after SLI diagnosis; however, evaluation of the effect on transplant-free survival time after HE development revealed that of the 23 patients with ALF, only one patient in the ALI group survived. Two patients in the ALI group and six in the SLI group required liver transplants. The mean transplant-free survival time after development of HE was 20 days (11 days in the ALI group and 21 days in the SLI group; Fig. 2) and there was no significant difference between the groups (Fig. 2). Two patients in the ALI group died and thus did not receive a liver transplant: one who was overage and another without an appropriate liver donor. Twelve patients in the ALF group died and thus did not undergo liver transplants; five were overage, four were absent of a liver donor, and three exhibited complications associated with contraindications for liver transplantation.

### Prediction of HE development in patients with SLI

Analysis of T-Bil, PT-INR, MELD score, and JHEP model using receiver operating characteristic curve (ROC) analysis is presented in Table 4. All data used in Table 4 were recorded at the time of SLI diagnosis. The areas under curve for T-Bil and PT-INR were 0.687 and 0.738, respectively. Those of the MELD score and JHEP model were 0.838 and 0.822, respectively, which were not significantly different.

**Table 4:** Predictive value for hepatic encephalopathy development in the 124 SLI patients.

	Cut-off value	AUROC	95% CI	Sensitivity	1-Specificity
T-Bil	12.6	0.687	0.582–0.792	0.6957	0.3069
PT-INR	1.95	0.738	0.602–0.875	0.6522	0.1584
JHEP model	33.76	0.822	0.734–0.909	0.913	0.4158
MELD score	16.70	0.838	0.754–0.922	0.9565	0.3960

*JHEP model* japan hepatic encephalopathy prediction model, *MELD* model of end stage liver disease, *PT-INR* prothrombin time international normalized ratio, *SLI* severe acute liver injury, *T-Bil* total bilirubin.

## Discussion

The present study revealed four major findings: (1) development of HE is a risk factor of poor prognosis in patients with SLI, (2) registration on the referral system before progression to SLI increases that time between SLI diagnosis and onset of HE, (3) registration on the referral system does not prolong transplant-free survival time after development of HE among patients who were registered with ALI, and (4) the MELD score and JHEP model can predict the development of HE in patients with SLI. We concluded that while early identification of SLI patients using the referral system increased the time from diagnosis of SLI to development of HE, it did not prolong the survival time after its development.

Although ALI is a self-limiting condition, the condition will progress to ALF in 1% of patients^17, 18^. Worldwide, the standard management for severe ALI is to transfer patients to a core center for intensive care^10^. The referral system that we implemented in 2004 resulted in a decrease in the number of patients with ALF due to initiation of preemptive therapy^16^. As our previous as well as the present study confirmed HE to be a risk factor for poor prognosis among patients with SLI on the referral system^19^, some patients in the ALI group were observed to develop HE, indicating that not all patients respond to preemptive therapy for HE. Thus, development of HE would be a surrogate marker for prognosis of the SLI patients and may be an indication for liver transplantation among patients with SLI who are registered on the referral system. Furthermore, MELD score and the JHEP model appear to have utility for the prediction of HE at the time of SLI diagnosis. Therefore, these calculations should be carried out at the time of progression to SLI for patients who are on the referral system.

Liver transplantation is an effective treatment for patients with ALF in whom the prognosis appears poor^12^; however, the waiting time to liver transplantation is considerable as several preparatory steps are required^20^. The present study revealed that registration on the referral system increased the time to onset of HE; therefore benefiting patients who require liver transplantation by allowing them to prepare for intensive care before the development of HE. Recently, several molecules have been reported as potential predictive markers of ALF prognosis^21,22^; these molecules may improve the predictive property of the referral system.

Combination therapy involving continuous hemodiafiltration (CHDF), plasma exchange, and the infusion of fresh frozen plasma is typically performed to support liver functions such as detoxification and supplementation of coagulation factors. In the core center of ALF treatment of Japan, patients are treated using a combination of these treatments^23^. Although patients were treated using preemptive therapy (Figs. 1, 2), enrollment on the referral system did not increase the survival time after onset of HE. These results indicate that the current standard therapy for ALF does not always improve clinical outcome; patients who do not respond to therapy have poor prognoses. It has recently been reported that the consciousness recovery rate improved with high flow (HF)-CHDF, which is performed with increased dialysate flow and filtrate rates^23^. However, HF-CHDF was not widely used because the procedure requires a specific piping system to provide increased dialysate flow; therefore, not all patients received HF-CHDF. A recent study published by us reported that a new mobile device for HF-CHDF was established^24^. As it does not require an existing pipe, HF-CHDF using this device can be performed at the bedside of ALF patients who may be in the intensive care unit. We aim to design a prospective study to confirm whether HF-CHDF via this mobile device can improve the prognosis of ALF patients on the referral system.

All ALF patients in the present study exhibited impaired biological dysfunction of hepatocytes, including impaired protein synthesis and detoxification, while deaths were found to be due to liver failure. To decrease the mortality of patients with ALF, functional recovery of the hepatocytes is needed. Liver regeneration does occur during ALF; however, this was found to be impaired in patients with ALF who died during the study period. Previous studies have reported that chronological changes in alpha fetoprotein maybe a marker for liver regeneration in patients with ALI, as well as a rodent model for liver injury^25–27^. In our previous study, patients who died exhibited decreased serum AFP levels and prothrombin activity during the study period^25^, while recovery of PT-INR following therapy was weak among patients who died^9^. We found that the livers of these patients showed impaired regenerative ability; liver transplantation (replacement of the mature organ) was often required. In future treatment of ALF patients, regaining the regenerative capacity may be a promising therapeutic target.

The present study has several limitations which should be acknowledged. First, the number of patients was relatively small; therefore, differences between the influence of specific etiologies on the prognosis of SLI remain unclear. We previously reported that the referral system does not decrease the rate of HE development among patients with SLI/ALF due to drug-induced liver disease^16^, nor does it improve the prognosis of patients with exacerbation of hepatitis B^3^; therefore, the clinical course of each etiology should be carefully observed. Second, the clinical course of the patients in this study does not reflect the natural course of SLI due to the selective population of the study. As some patients progressed to ALF despite preemptive therapy, we suspect that there will be patients who do not respond to the therapy in this population. Although there may have been a selection bias in the study population, the development of HE is reasonable as a prognostic factor for patients with ALI. Thus, the results of this study will be useful for identifying patients with SLI with a poor prognosis to decide treatment strategies. Finally, the population of the present study are from an approximate homogenic racial community; therefore, the findings may not be generalized to other populations. To generalize these findings, further study within heterogenic racial communities are required.

## Methods

### Subjects

We implemented the referral system for patients with ALI in 2004. Parts of the results have previously been reported by us^3,7,9,16,25,28^. The present observational prospective cohort study was registered in University hospital Medical Information Network (UMIN) in 2015 (UMIN000016568). All study protocol was approved by the Institutional Review Board of Iwate Medical University (H20-36). The study was performed in accordance with the relevant guidelines and regulations. Informed consent was obtained from all participants or the parents/legal guardians of minors. Criteria for registration on the referral system included an ALI diagnosis (AST > 200 IU/L or ALT > 300 IU/L) and PT activity ≤ 80%. Our database included 730 consecutive patients who were referred to our department for evaluation of liver dysfunction from December 2004–2018. ALI, SLI, and ALF were defined as follows: ALI referred to elevated liver enzyme levels (AST > 200 U/L or ALT > 300 U/L) in patients without chronic liver disease, SLI referred to ALI with coagulopathy (PT-INR ≥ 1.5), and ALF referred to SLI with HE. The etiology of these patients included either hepatitis or non-hepatitis, while disease types included ALI, SLI, or ALF. For the present study, we enrolled all patients who were diagnosed with SLI upon registration on the database, or who satisfied the SLI criteria during observation. The inclusion criteria of the study were as follows: the absence of chronic hepatitis or liver cirrhosis, a diagnosis of ALI due to hepatitis, sufficient data for analysis, and prolonged PT-INR ≥ 1.5 or PT activity ≤ 40%. Evaluation of etiology was based on previously described criteria^3^. The exclusion criterion was a diagnosis of ALI due to non-hepatitis (malignancy, ischemic hepatitis, congestive liver, or acute fatty liver of pregnancy). Because established treatment for SLI, except for autoimmune hepatitis, was absent and the present study was observational, all patients were treated using at least general therapy. Once the patients progressed to ALF, artificial liver support such as hemodiafiltration was performed for support of impaired detoxification.

### Laboratory data

Plasma PT and serum levels of ALT, AST, Cre, and T-Bil were determined using an autoanalyzer (JCA-BM2250; JEOL, Tokyo, Japan). Data were collected at ALF diagnosis.

### Japan hepatic encephalopathy prediction model

The JHEP value was calculated using the following formula^8^: *P* = 100/(1 + eλ), where λ = [0.692 log_e_ (1 + T-Bil)] − 0.065 PT + [1.388 $\times$ age in years] + [0.868 $\times$ etiology] − 1.156. In this formula, the units of T-Bil are mg/dL; PT is percentage. Age is given as 1 for patients older than 50 years, while “etiology” is given the value of 1 or 0; 1 in the case of non-acetaminophen-induced liver injury flaring up due to type B hepatitis, autoimmune hepatitis, or an unknown cause, and 0 for other causes.

### Model of end-stage liver disease score

The Model of End-stage Liver Disease (MELD) score was calculated using the following formula, based on the results of the hematological examination^5^: MELD = 9.57 loge [Cre] + 3.78log_e_ [T-Bil] + 11.20log_e_ [PT‐INR] + 6.43. In this formula, the units of Cre and T-Bil are mg/dL.

### Statistical analysis

The primary endpoint of this study was either death or liver transplantation during hospitalization. Data are presented as mean values with 95% confidential interval or range (minimum–maximum). All analyses were performed using SPSS version 17.0 (SPSS Inc., Chicago, IL, USA) or JMP Pro 13 (SAS Institute, Cary, NC, USA). The chi-squared test, Student’s t-test, Wilcoxon/Kruskal–Wallis test, and Mann–Whitney U test were used to evaluate the statistical significance of the results; a two-sided *p *value of < 0.05 was considered statistically significant. Binomial logistic regression analysis was performed to identify factors associated with the primary endpoint in patients with SLI; survival or deceased/requiring liver transplantation were used as the objective variables. Laboratory data (ALT, Cre, T-Bil, and PT-INR), sex, age, SLI at registration, and the presence of HE were used as explanatory variables. The contribution of explanatory variables to the objective variable was expressed as the odds ratio. Kaplan–Meier analysis was used to evaluate the cumulative rate of events. Comparison of the cumulative rate between two groups was carried out using the log-rank test. An ROC was used to assess the diagnostic performance of HE development. The cut-off values were estimated by using the area under the ROC (AUROC) method.

## Supplementary information

Supplementary Information.
