# Supplementary material for: Early identification using the referral system prolonged the time to onset for hepatic encephalopathy after diagnosing severe acute liver injury
Source: Sci Rep. 2020 Oct 14;10:17280. doi: 10.1038/s41598-020-74466-2 (PMC7560720; doi:10.1038/s41598-020-74466-2)
Supplement: Supplementary file 1 — Supplementary Information. [file 41598_2020_74466_MOESM1_ESM.docx]

*Early identification using the referral system prolonged the time to onset for hepatic encephalopathy after diagnosing severe acute liver injury*

*Keisuke KAKISAKA M.D., Ph.D.,* *Yuji SUZUKI M.D., Ph.D., Hiroaki ABE M.D., Takuya WATANABE M.D., Kenji YUSA M.D., Hiroki SATO M.D., and Yasuhiro TAKIKAWA M.D., Ph.D.*

Supplemental Table 1 Multivariate analysis about prognosis using by sex, presence of hepatic encephalopathy, age, creatinine, prothrombin, total bilirubin aspartate aminotransferase and alanine aminotransferase in the 124 SLI patients

|  | Odds | 95% CI | p value |
| --- | --- | --- | --- |
| Sex (F) | 0.67 | 0.14 – 3.14 | 0.6091 |
| Coma (+) | 195.71 | 14.28 – 2681.48 | <0.0001 |
| Age | 1.04 | 0.98 – 1.09 | 0.1763 |
| Cre | 5.15 | 0.38 – 70.06 | 0.2181 |
| PT-INR | 1.61 | 0.41 – 6.35 | 0.4939 |
| T-Bil | 1.01 | 0.95 - 1.08 | 0.6938 |
| ALT | 1.00 | 0.99 – 1.01 | 0.2316 |
| AST | 1.00 | 0.99 – 1.01 | 0.7135 |
